# Prevalence and correlates of anxiety and depressive symptoms among adolescents aged 10–19 years in six sub-Saharan African countries, China and India: A cross-sectional study

**DOI:** 10.1371/journal.pmen.0000479

**Published:** 2025-10-27

**Authors:** Shraddha Bajaria, Innocent Yusufu, Innocent B. Mboya, Addis Eyeberu, Yadeta Dessie, Nega Assefa, Sachin Shinde, Rutuja Patil, Kun Tang, Ayoade Oduola, David Guwatudde, Japhet Killewo, Frank Mapendo, Mashavu Yussuf, Amani Tinkasimile, Adom Manu, Ali Sie, Yemane Berhane, Mosa Moshabela, Lina Nurhussein, Mary Mwanyika Sando, Wafaie Fawzi

**Affiliations:** 1 Africa Academy for Public Health, Dar es Salaam, Tanzania; 2 Department of Epidemiology and Biostatistics, School of Public Health, KCMC University, Moshi, Tanzania; 3 School of Nursing and Midwifery, College of Health and Medical Sciences, Haramaya University, Ethiopia; 4 School of Public Health, College of Health and Medical Sciences, Haramaya University, Ethiopia; 5 Department of Global Health and Population, Harvard T.H. Chan School of Public Health, Boston, Massachusetts, United States of America; 6 KEM Hospital Research Center, Pune, India; 7 Vanke School of Public Health, Tsinghua University, China; 8 University of Ibadan Research Foundation, University of Ibadan, Ibadan, Nigeria; 9 Department of Epidemiology and Biostatistics, Makerere University School of Public Health, Kampala, Uganda; 10 Department of Epidemiology and Biostatistics, Muhimbili University of Health and Allied Sciences, Dar es Salaam, Tanzania; 11 Department of Population Health, School of Public Health, University of Ghana, Accra, Ghana; 12 Nouna Health Research Center, Nouna, Burkina Faso; 13 Addis Continental Institute of Public Health, Addis Ababa, Ethiopia; 14 University of KwaZulu-Natal, Pietermaritzburg, South Africa; 15 Department of Epidemiology, Harvard T.H. Chan School of Public Health, Boston, Massachusetts, United States of America; 16 Department of Nutrition, Harvard T.H. Chan School of Public Health, Boston, Massachusetts, United States of America; FSBSI Scientific Research Institute of Neurosciences and Medicine: FGBNU Naucno-issledovatel'skij institut nejronauk i mediciny, RUSSIAN FEDERATION

## Abstract

Although adolescence (10–19 years) signifies a vulnerable phase for the onset of depression and anxiety, research on the symptoms of these mental health conditions is scarce in low- and middle-income countries. This study examined the prevalence and correlates of anxiety and depressive symptoms among adolescents in six sub-Saharan African countries (SSA), China, and India, where these conditions are often underdiagnosed, highlighting the need for evidence to support culturally appropriate interventions. We analyzed cross-sectional survey data from 9,849 adolescents in Burkina Faso, Ethiopia, Ghana, Nigeria, Tanzania, Uganda, China, and India. The Patient Health Questionnaire (PHQ-4) assessed the prevalence of anxiety and depressive symptoms. Log-linear regression models estimated the relative risk and 95% confidence intervals (CI) for the correlates of anxiety and depressive symptoms. The overall prevalence of anxiety and depressive symptoms was 11.3% (95%CI 10.6-11.9%) and 9.9% (95%CI 9.3-10.5%), respectively. Prevalence varied significantly across countries (p-value<0.001), with high rates of anxiety and depressive symptoms found in China (26.3%, 17.0%), Ghana (17.6%, 19.6%), and Nigeria (13.2%, 13.7%). Adolescents aged 15–19 years, females, with low socioeconomic status (SES), who visited a health facility, those with a history of alcohol use, or those who experienced multiple serious injuries in the past year, had an increased risk of anxiety and depressive symptoms. Adolescents with regular physical activity had a reduced risk of anxiety and depressive symptoms. Anxiety and depressive symptoms are common among adolescents in China, Ghana, and Nigeria, but relatively rare in India and Ethiopia. These symptoms of mental health issues are associated with socio-demographic characteristics and behaviors. These findings suggest a need for policies promoting healthy lifestyles and programs to reduce risks among adolescents through physical activity, integrated mental health screening, and substance use prevention, particularly for older adolescents, females, those with a low SES, and those with a history of substance use.

## Author summary

Adolescents, individuals aged 10–19 years, in low- and middle-income countries are at high risk of mental health disorders such as anxiety and depression but are often underdiagnosed. This study examined the prevalence and correlates of anxiety and depressive symptoms among nearly 10,000 adolescents in Burkina Faso, Ethiopia, Ghana, Nigeria, Tanzania, Uganda, China, and India. Findings indicate that about one in ten adolescents had anxiety, and a tenth had depressive symptoms. Individuals most at risk for these mental health symptoms were older adolescents aged 15–19 years, girls, from low-income households, who had visited a health facility, with a history of multiple physical injuries, and those who consumed substances such as alcohol. However, regular physical activity for five or more days per week reduced the risk of these symptoms. Policies and programs should promote healthy lifestyles among adolescents through regular physical activity, integrated mental health screening, and substance use prevention within school and out-of-school settings.

## Introduction

Mental health disorders are among the leading causes of illness and disability among adolescents [1]. Globally, approximately one in seven adolescents experiences a mental health disorder, accounting for about 13% of the global burden of disease in this age group [1]. In 2021, an estimated 3.6% of adolescents aged 10–14 years and 4.6% of those aged 15–19 years experienced anxiety, while 1.1% and 2.8% experienced depression, respectively [1]. The prevalence of anxiety and depression is even higher among adolescents in low- and middle-income countries (LMICs), where adolescents constitute a majority of the population [2]. In 2023, the prevalence of anxiety and depression among adolescents in LMICs was 5.5% and 3.1% respectively [3]. In sub-Saharan Africa (SSA), one out of every seven adolescents (14%) experience a mental health disorder [1]. In India, the prevalence of mental disorders among 13–17-year-olds was about 7% [4], while in China, the prevalence of anxiety symptoms was 27%, and 25% of the adolescents had depressive symptoms [5].

In LMICs, adolescents’ exposure to adverse conditions such as high burden of HIV/AIDS, violence, poverty, food insecurity, low socio-economic status, stigma, and lack of access to quality healthcare increases their vulnerability to mental health disorders [2]. A cross-sectional study in six SSA countries reported a high risk of depressive symptoms among girls, older adolescents (18–19 years), and adolescents experiencing bullying, violence, and food insecurity [6]. This and other similar studies show that the correlates of depression and anxiety are similar across LMICs [3,6,7]. In addition, anxiety and depression have an impact on adolescents’ overall health [7]. Evidence shows that low educational achievement [7], suicidal ideation and attempts [8], and poor physical health are some of the negative consequences of anxiety and depressive symptoms in adolescence.

Despite being a leading cause of morbidity and mortality in LMICs [9], data systems and initiatives for mental health remain under-prioritized. There remains a dearth of evidence for the true burden of mental health among adolescents in LMICs [10]; hence, fewer health policies and large-scale interventions are available. Global initiatives, such as the Comprehensive Mental Health Action Plan 2013–2030 and the Mental Health Gap Action Programme (mhGAP), are aimed at promoting mental health and well-being for all people, to prevent mental health conditions among at-risk populations, especially in LMICs [11]. However, the implementation of these action plans remains slow, especially in Africa.

While there has been emerging interest in country-specific research on adolescent mental health, population-based multi-site studies to investigate the burden and correlates of mental health disorders among adolescents in sub-Saharan Africa (SSA), China, and India are limited [12]. Most previous studies explored anxiety and depressive symptoms within school settings and have mainly focused on older adolescents and girls [10]. This study assessed the prevalence and correlates of anxiety and depressive symptoms among in-school and out-of-school adolescents aged 10–19 years in six SSA countries, China, and India.

## Methods

### Ethics statement

Ethical approval to conduct the survey was obtained from the Institutional Review Board (IRB) of the Harvard T.H. Chan School of Public Health (IRB21–0696) and the Ethical Review Boards at and Ministry of Health in Burkina Faso, Tsinghua University in China, the College of Health and Medical Sciences, Haramaya University in Ethiopia, the Ghana Health Service in Ghana, the KEM Hospital Research Centre in India, the University of Ibadan in Nigeria, the National Institute for Medical Research in Tanzania, and Makerere University School of Public Health in Uganda. Before the survey, written informed consent was obtained from participants aged ≥18, while parental consent and participant assent were obtained from minor participants (<18).

### Study design, setting, and population

We used cross-sectional survey data from six countries in sub-Saharan Africa and two in Asia, as part of the African Research, Implementation Science, and Education (ARISE) Network’s adolescent health and well-being longitudinal study [13]. The ARISE Network has developed a core set of priority indicators relevant to adolescent health, which were used to collect data for this study [13]. Data analysis focused on the first of two rounds of repeated surveys conducted at the household level in Nouna, Burkina Faso; Harar, Ethiopia; Shai Osudoku/Ningo Prampram, Ghana; Dar es Salaam, Tanzania; Iganga, Uganda; and Pune, India; and school-based in Funan County, China, and Ibadan, Nigeria in 2021 and 2022, among adolescent girls and boys aged 10–19 years.

Adolescents were randomly sampled from existing population cohorts maintained by Health and Demographic Surveillance Systems (HDSSs), which gather longitudinal data on dynamic cohorts of the total population in many developing countries. For this study, household or school-based sampling was used at each study site to randomly select eligible adolescents within the households or schools covered by the HDSS, ensuring accurate representation of the adolescent population in the community. At baseline, approximately 1200 adolescents were recruited at each study site. Sample size was estimated using the prevalence of anemia in adolescents, which was the primary outcome in the main study, ranging from 20 to 60%, assuming 1000 adolescents per site, and accounting for a 20% loss to follow-up. Further information about the ARISE Network, the study design, and sampling strategy is available in detail elsewhere [13].

Adolescents were included in the study if (i) they were between the ages of 10–19 years; (ii) signed consent from a parent or guardian if under 18 years of age; (iii) provided an assent to participate if <18 years of age; and (iv) signed consent if 18–19 years of age. Adolescents were excluded from the study if they could not converse fluently in English or a site-specific language, or if they were visitors or temporary residents unlikely to remain in the study area for the duration of the study.

### Data collection

Trained interviewers collected data in-person at households and schools across all sites using the ARISE Network developed survey instrument on key adolescent health indicators covering six domains of adolescent health. These included physical health, nutrition, mental health, substance use, sexual and reproductive health, and health services utilization [13]. The instrument was designed in English and subsequently translated into and administered using the site-specific local languages. The data collection exercise across all sites was implemented between November 2021 and July 2022.

### Measurements

The primary outcomes in this study were anxiety and depressive symptoms, assessed using the four-item Patient Health Questionnaire (PHQ-4). The tool has been validated to screen for depression and anxiety symptoms in other settings [14]. The first two items (PHQ-2) screened for depressive symptoms through the following questions: 1) “Over the last two weeks, how often have you been bothered by feeling down, depressed, or hopeless?” and 2) “Over the last two weeks, how often have you been bothered by little interest or pleasure in doing things?” The remaining two questions screened for generalized anxiety disorder (GAD-2). The questions were: 3) “Over the last two weeks, how often have you been bothered by feeling nervous, anxious, or on edge?” and 4) “Over the last two weeks, how often have you been bothered by not being able to stop or control worrying?”. Responses to these questions were collected on a 4-point Likert scale: not at all (0), several days [1], more than half the days [2], and nearly every day [3]. The scores for each subscale range from 0 to 6. For each outcome, a score of 3 or higher has been shown to have high sensitivity and specificity for detecting symptoms of depression and anxiety [14].

Other independent variables were socio-demographic and behavioral characteristics, and anthropometrics. Socio-demographic characteristics included age (10–14 years, 15–9 years), sex (male, female), education level (none/primary, secondary or higher), mobile phone ownership, and having health insurance. Behavioral characteristics included a history of substance use, particularly alcohol use and cigarette smoking, physical activity (number of days the participant had been physically active in the past 7 days), and ever having sexual intercourse. Anthropometric measurements included height, weight, and body mass index (BMI). BMI was classified using the WHO growth standards for 5–19-year-olds. A BMI-for-age z-score of <-2 standard deviations (SD) was classified as underweight, ≥ -2 to +1 as normal weight, >+1 as overweight, and>+2 as obese.

Additionally, subjective social status (SSS) was measured using the MacArthur scale of SSS, a one-item validated measure assessing a person’s perceived rank of social status [15]. For this, respondents viewed a drawing of a 10-rung ladder, the top indicating people who are best off, i.e., those who have the most money, the most education, and the most respected jobs. The bottom indicates the people who are the worst off, i.e., who have the least money, the least education, and the least respected job or no job. Respondents were asked to indicate which rung best represents their standing on the ladder, with responses ranging from 1 to 10. Scores of 0–3, 4–7, and 8–10 were classified as low, middle, and high SSS, respectively.

### Statistical analysis

Data analysis was performed using Stata 17.0 software (StataCorp, College Station, Texas 77845 USA). Participant characteristics were summarized using frequencies, proportions, means, and standard deviations as appropriate and by country. Differences in the prevalence of anxiety and depressive symptoms by countries were assessed using the Chi-squared test. Log-linear regression models (i.e., the generalized linear regression models with Poisson family, log-link function, and robust variance estimator) were used to estimate the adjusted relative risk (RR) and their 95% confidence intervals (CI) for the correlates of anxiety and depressive symptoms, using pooled and country-specific data. All statistical tests were two-tailed at 5% threshold level.

### Inclusivity in global research

Additional information regarding the ethical, cultural, and scientific considerations specific to inclusivity in global research is included in the S1 Checklist.

## Results

### Characteristics of adolescents overall and by country

Of the 9,849 adolescents included in the survey, 57.1% were aged 10–14 years, and 52.2% were females. Most adolescents (86.7%) attended a school/educational institute at the time of the survey. About 29% of adolescents owned a mobile phone, and 18% had worked in the past year, and a fifth (19.5%) perceived that they had a low social status. Only 21.1% of the adolescents had health insurance, and 10.4% ever had sexual intercourse. Additionally, 3.1% of adolescents had a history of cigarette smoking, and 15.6% had consumed alcohol in their lifetime. Almost half (51.8%) of all adolescents were engaged in regular physical activity for 5–7 days a week. The prevalence of underweight and overweight/obesity was 8.8% and 20.5%, respectively (**Table 1**).

**Table 1:** Characteristics of adolescents in six SSA countries, China, and India, overall and by the study site.

Characteristics	Total	Burkina Faso	Ethiopia	Ghana	Nigeria	Tanzania	Uganda	China	India
**n (%)**	**n (%)**	**n (%)**	**n (%)**	**n (%)**	**n (%)**	**n (%)**	**n (%)**	**n (%)**
**Age (years)**									
10-14	5621 (57.1)	703 (58.5)	382 (**31.8)**	705 (56.6)	415 (**32.8**)	641 (**53.4**)	776 (**64.7**)	1285 (**99.5**)	714 (57.3)
15-19	4228 (42.9)	499 (41.5)	818 (**68.2**)	540 (43.4)	849 (**67.2**)	560 (**46.6**)	424 (**35.3**)	6 (**0.5**)	532 (42.7)
**Sex**									
Male	4710 (47.8)	679 (**56.5**)	462 (**38.5**)	571 (45.9)	506 (**40.0**)	524 (**43.6**)	595 (49.6)	699 (**54.1**)	674 (**54.1**)
Female	5139 (52.2)	523 (**43.5**)	738 (**61.5**)	674 (54.1)	758 (**60.0**)	677 (**56.4**)	605 (50.4)	592 (**45.9**)	572 (**45.9**)
**Currently in school/ college/ vocational institute** (Yes)	7407 (86.7)	714 (**59.4**)	1155 (**96.3**)	1060 (85.8)	1234 (**98.2**)	989 (**82.3**)	1094 (**91.2**)	–	1161 (**93.3**)
**Education level**									
None/Primary	5941 (60.3)	950 (**79.0**)	405 (**33.8**)	819 (**65.8**)	185 (**14.6**)	761 (**63.4**)	1006 (**83.8**)	1291 (**100.0**)	524 (**42.1**)
Secondary or higher	3908 (39.7)	252 (**21.0**)	795 (**66.3**)	426 (**34.2**)	1079 (**85.4**)	440 (**36.6**)	194 (**16.2**)	–	722 (**57.9**)
**Owned a mobile phone** (Yes)	2826 (28.7)	245 (**20.4**)	728 (**60.7**)	320 (**25.7**)	663 (**52.5**)	255 (**21.2**)	149 (**12.4**)	466 (**36.1**)	–
**Engaged in any work past year** (Yes)	1733 (17.6)	478 (**39.8**)	132 (**11.0**)	253 (**20.3**)	235 (**18.6**)	169 (14.1)	256 (**21.3**)	–	210 (16.9)
**MacArthur Scale of Subjective Social Status**									
Low	1923 (19.5)	334 (**27.8**)	203 (**16.9**)	274 (22.0)	193 (**15.3**)	244 (20.3)	375 (**31.3**)	236 (18.3)	64 (**5.1**)
Middle	7198 (73.1)	820 (**68.2**)	962 (**80.2**)	791 (**63.5**)	968 (**76.6**)	916 (**76.3**)	804 (**67.0**)	971 (75.2)	966 (**77.5**)
High	728 (7.4)	48 (**4.0**)	35 (**2.9**)	180 (**14.5**)	103 (8.1)	41 (**3.4**)	21 (**1.8**)	84 (6.5)	216 (**17.3**)
**Visited primary care clinic past year** (Yes)	1394 (14.2)	225 (**18.7**)	57 (**4.8**)	126 (**10.1**)	71 (**5.6**)	114 (**9.5**)	278 (**23.2**)	325 (**25.2**)	198 (15.9)
**Visited a hospital past year** (Yes)	2633 (26.7)	107 (**8.9**)	252 **(21.0**)	273 (**21.9**)	326 (25.8)	554 (**46.1**)	210 (**17.5**)	392 (**30.4**)	519 (**41.7**)
**Visited a drug pharmacy or store past year** (Yes)	2390 (24.3)	99 (**8.2**)	25 (**2.1**)	564 (**45.3**)	405 (**32.0**)	141 (**11.7**)	415 (**34.6**)	549 (**42.5**)	192 (**15.4**)
**Have health insurance**									
Yes	2083 (21.1)	14 (**1.2**)	300 (**25.0**)	914 (**73.4**)	70 (**5.5**)	142 (**11.8**)	2 (**0.2**)	352 (**27.3**)	289 (23.2)
No	6687 (67.9)	1162 (**96.7**)	707 (**58.9**)	322 (**25.9**)	1102 (**87.2**)	1041 (**86.7**)	1198 (**99.8**)	222 (**17.2**)	933 (**74.9**)
Don’t know	1079 (11.0)	26 (**2.2**)	193 (**16.1**)	9 (**0.7**)	92 (**7.3**)	18 (**1.5**)	–	717 (**55.5**)	24 (**1.9**)
**Ever smoked cigarettes** (Yes)	307 (3.1)	43 (3.6)	91 (**7.6**)	48 (3.9)	16 (**1.3**)	48 (4.0)	8 (**0.7**)	50 (3.9)	3 (**0.2**)
**Ever drank alcohol** (Yes)	1533 (15.6)	326 (**27.1**)	200 (16.7)	465 (**37.3**)	166 (**13.1**)	85 (**7.1**)	33 (**2.8**)	256 (**19.8**)	2 (**0.2**)
**Ever had sexual intercourse with a girl, woman, boy, or man** (Yes)	1020 (10.4)	190 (**15.8**)	165 (**13.8**)	248 (**19.9**)	56 (**4.4**)	165 (**13.7**)	166 (**13.8**)	24 (**1.9**)	6 (**0.5**)
**Seriously injured two or more times in the past year** (Yes)	1815 (18.4)	42 (**3.5**)	6 (**0.5**)	140 (**11.2**)	243 (19.2)	18 (**1.5**)	62 (**5.2**)	1291 (**100.0**)	13 (**1.0**)
**Number of days physically active past week**									
None	1121 (11.4)	132 (11.0)	51 (**4.3**)	20 (**1.6**)	61 (**4.8**)	251 (**20.9**)	108 (**9.0**)	94 (**7.3**)	404 (**32.4**)
1-4 days	3628 (36.8)	508 (**42.3**)	450 (37.5)	139 (**11.2**)	378 (**29.9**)	426 (35.5)	660 (**55.0**)	643 (**49.8**)	424 (**34.0**)
5-7 days	5100 (51.8)	562 (**46.8**)	699 (**58.3**)	1086 (**87.2**)	825 (**65.3**)	524 (**43.6**)	432 (**36.0**)	554 (**42.9**)	418 (**33.5**)
**Body mass index (kg/m**^**2**^)									
Normal	6964 (70.7)	1045 (**86.9**)	941 (**78.4**)	940 (**75.5**)	822 (**66.0**)	957 (**79.7**)	1026 (**85.5**)	192 (**14.9**)	1041 (**82.4**)
Underweight	863 (8.8)	107 (8.9)	121 (10.1)	77 (**6.2**)	258 (20.7)	105 (8.7)	54 (**4.5**)	3 (**0.2**)	138 (**10.9**)
Overweight/obese	2022 (20.5)	50 (**4.2**)	138 (**11.5**)	228 (18.3)	166 (**13.3**)	139 (**11.6**)	120 (**10.0**)	1096 (**84.9**)	85 (**6.7**)
**Total**	**9849**	**1202 (12.2)**	**1200 (12.2)**	**1245 (12.6)**	**1264 (12.8)**	**1201 (12.2)**	**1200 (12.2)**	**1291 (13.1)**	**1246 (12.7)**

The Chi-squared test comparing the respondent characteristics across study countries yielded statistically significant results for all variables (p < 0.001). Bolded proportions are statistically significantly different from the total (overall) proportions (p < 0.05, Wald Test for proportions).

Across countries, older adolescents (15–19 years) constituted the highest proportion in Ethiopia (68.2%) and Nigeria (67.2%), while in China, nearly all (99.5%) were younger adolescents (10–14 years). Two-thirds (66.3%) of the adolescents in Ethiopia and 85.4% in Nigeria had attained secondary or higher education. A higher proportion (39.8%) of adolescents in Burkina Faso worked in the past year, compared to other countries. The proportion of perceived low social status was highest in Uganda (31.3%), Burkina Faso (27.8%), and Tanzania (20.3%). The prevalence of alcohol consumption was highest in Ghana (37.3%), followed by Burkina Faso (27.1%), China (19.8%), and Ethiopia (16.7%). The majority (87.2%) of the adolescents from Ghana had been physically active 5–7 days a week preceding the survey, followed by 65.3% in Nigeria, and 58.3% in Ethiopia. A higher proportion (84.9%) of adolescents from China were overweight or obese (**Table 1**).

### Prevalence of anxiety and depressive symptoms by country

The overall prevalence of anxiety and depressive symptoms among adolescents was 11.3% (95%CI 10.6-11.9%) and 9.9% (95%CI 9.3-10.5%), respectively, and prevalence differed significantly by country (p < 0.001). Adolescents in China had the highest prevalence of anxiety symptoms (26.3%, 95%CI 23.9-28.7%), while depressive symptoms were the most prevalent among adolescents in Ghana (19.6%, 95%CI 17.5-21.9%). India and Ethiopia had the lowest prevalence of these outcomes compared to other countries. The prevalence of anxiety symptoms was significantly higher among females (12%) compared to males (10.5%), while the proportions were similar across age categories and school enrollment status. Other adolescent characteristics showed statistically significant associations with anxiety symptoms. Conversely, older adolescents aged 15–19 years had a statistically significantly higher prevalence of depressive symptoms (10.6%) than younger adolescents aged 10–14 years (9.3%). The prevalence of depressive symptoms was consistent across sex, school enrollment status, educational attainment, and physical activity, though significant associations were observed with other characteristics of adolescents (**Table 2**).

**Table 2:** Prevalence of anxiety and depressive symptoms among adolescents in six SSA countries, China, and India across participant characteristics.

Characteristics	Anxiety (Yes) n (%)	P-value*	Depression (Yes) n (%)	P-value*
**Age (years)**		0.716		0.03
10-14	638 (11.4)		524 (9.3)	
15-19	470 (11.1)		450 (10.6)	
**Sex**		0.019		0.257
Male	493 (10.5)		449 (9.5)	
Female	615 (12.0)		525 (10.2)	
**Currently in school/ college/ vocational institute**		0.49		0.208
Yes	660 (**8.9**)		642 (**8.7**)	
No	108 (9.5)		111 (9.8)	
**Education level**		0.005		0.158
None/Primary	711 (12.0)		608 (10.2)	
Secondary or higher	397 (**10.2**)		366 (9.4)	
**Owned a mobile phone**		<0.001		<0.001
Yes	406 (**14.4**)		338 (**12.0**)	
No	702 (**10.0**)		636 (**9.1**)	
**Engaged in any work past year**		0.024		<0.001
Yes	222 (**12.8**)		214 (**12.3**)	
No	886 (10.9)		760 (9.4)	
**MacArthur Scale of Subjective Social Status**		<0.001		<0.001
Low	284 (**14.8**)		262 (**13.6**)	
Middle	758 (10.5)		647 (**9.0**)	
High	66 (9.1)		65 (8.9)	
**Visited a primary care clinic past year**		0.021		<0.001
No	926 (11.0)		800 (9.5)	
Yes	182 (**13.1**)		174 (**12.5**)	
**Visited a hospital past year**		<0.001		0.003
No	765 (10.6)		675 (9.4)	
Yes	343 (**13.0**)		299 (**11.4**)	
**Visited a drug pharmacy or store past year**		<0.001		<0.001
No	709 (**9.5**)		589 (**7.9**)	
Yes	399 (**16.7**)		385 (**16.1**)	
**Have health insurance**		<0.001		<0.001
Yes	289 (**13.9**)		273 (**13.1**)	
No	619 (**9.3**)		582 (**8.7**)	
Don’t know	200 (**18.5**)		119 (11.0)	
**Ever smoked cigarettes**		0.022		0.001
Yes	47 (**15.3**)		47 (**15.3**)	
No	1061 (11.1)		927 (9.7)	
**Ever dank alcohol**		<0.001		<0.001
Yes	266 (**17.4**)		255 (**16.6**)	
No	842 (**10.1**)		719 (**8.6**)	
**Ever had sexual intercourse with a girl, woman, boy, or man**		<0.001		<0.001
No	939 (10.6)		818 (9.3)	
Yes	169 (**16.6**)		156 (**15.3**)	
**Seriously injured two or more times in the past year**		<0.001		<0.001
No	672 (**8.4**)		659 (**8.2**)	
Yes	436 (**24.0**)		315 (**17.4**)	
**Number of days physically active past week**		<0.001		0.078
None	121 (10.8)		106 (9.5)	
1-4 days	482 (**13.3**)		391 (10.8)	
5-7 days	505 (**9.9**)		477 (9.4)	
**Body mass index (kg/m**^**2**^)		<0.001		<0.001
Normal	663 (**9.5**)		637 (**9.1**)	
Underweight	67 (**7.8**)		64 (**7.4**)	
Overweight/obese	378 (**18.7**)		273 (**13.5**)	
**Country**		<0.001		<0.001
Tanzania	60 (**5.0**)		61 (**5.1**)	
Ethiopia	35 (**2.9**)		23 (**1.9**)	
Burkina Faso	108 (**9.0**)		84 (**7.0**)	
Uganda	154 (12.8)		157 (**13.1**)	
Ghana	219 (**17.6**)		244 (**19.6**)	
India	26 (**2.1**)		13 (**1.0**)	
Nigeria	167 (**13.2**)		173 (**13.7**)	
China	339 (**26.3**)		219 (**17.0**)	
**Total**	1108 (11.2)		974 (9.9)	

### Correlates of anxiety symptoms

Older adolescents than younger adolescents and females than males had a significantly higher risk of experiencing anxiety symptoms. Those who engaged in any work in the past year, had low perceived social status and visited a hospital in the past year, also had a higher risk of experiencing anxiety symptoms than their counterparts. Ever drinking alcohol, and being seriously injured multiple (≥ 2) times in the past year also increased the risk of anxiety symptoms. Being physically active for 5–7 days in the past week was associated with a 44% decreased risk of experiencing anxiety symptoms. Compared to adolescents in Tanzania, the study found that those from Burkina Faso, Uganda, Ghana, India, and China had strong positive associations with anxiety symptoms. Conversely, adolescents from Ethiopia and Nigeria showed inverse protective associations, demonstrating their lower likelihood of having anxiety symptoms than those in Tanzania (**Table 3**).

**Table 3:** Correlates of anxiety and depressive symptoms among adolescents aged 10-19 years in six sub-Saharan African countries, China, and India.

Characteristics	Anxiety		Depressive symptoms	
	RR (95% CI)	p-value	RR (95% CI)	p-value
**Age** (15–19 years)	1.35 (1.14, 1.60)	0.001	1.39 (1.17, 1.67)	<0.001
**Sex** (Female)	1.23 (1.10, 1.38)	<0.001	1.13 (1.00, 1.28)	0.05
**Education level** (Secondary or higher)	1.29 (1.09, 1.54)	0.004	1.09 (0.91, 1.30)	0.35
**Engaged in any work past year** (Yes)	1.28 (1.10, 1.50)	0.002	1.24 (1.06, 1.45)	0.01
**MacArthur Scale of Subjective Social Status** (ref: High)				
Low	1.53 (1.20, 1.95)	0.001	1.43 (1.12, 1.83)	<0.001
Middle	1.19 (0.95, 1.50)	0.14	1.07 (0.85, 1.35)	0.61
**Visited primary care clinic past year** (Yes)	0.98 (0.85, 1.14)	0.82	1.19 (1.02, 1.38)	0.03
**Visited a hospital past year** (Yes)	1.31 (1.17, 1.48)	<0.001	1.36 (1.20, 1.55)	<0.001
**Visited a drug pharmacy or store past year** (Yes)	1.12 (0.99, 1.26)	0.05	1.30 (1.15, 1.47)	<0.001
**Have health insurance** (ref: No)				
Yes	0.96 (0.81, 1.14)	0.63	0.94 (0.78, 1.13)	0.49
Don’t know	0.88 (0.73, 1.06)	0.17	0.71 (0.56, 0.89)	0.003
**Ever smoked cigarettes** (Yes)	1.15 (0.87, 1.51)	0.32	1.32 (0.99, 1.75)	0.05
**Ever drank alcohol** (Yes)	1.18 (1.03, 1.36)	0.02	1.28 (1.09, 1.48)	0.001
**Ever had sexual intercourse with a girl, woman, boy, or man** (Yes)	1.19 (0.99, 1.43)	0.05	1.07 (0.89, 1.30)	0.47
**Seriously injured multiple (≥** 2**) times past year** (Yes)	1.45 (1.18, 1.77)	<0.001	1.35 (1.11, 1.65)	0.003
**Number of days physically active past week** (ref: None)				
1-4 days	0.81 (0.68, 0.98)	0.03	0.76 (0.63, 0.93)	0.01
5-7 days	0.56 (0.46, 0.68)	<0.001	0.54 (0.44, 0.66)	<0.001
**Body mass index** (ref: Normal)				
Underweight	1.15 (0.91, 1.47)	0.24	1.13 (0.89, 1.44)	0.31
Overweight/obesity	1.03 (0.88, 1.22)	0.69	1.02 (0.86, 1.21)	0.84
**Country** (ref: Tanzania)				
Ethiopia	0.61 (0.41, 0.92)	0.019	0.44 (0.27, 0.71)	0.001
Burkina Faso	2.01 (1.46, 2.76)	<0.001	1.47 (1.05, 2.07)	0.024
Uganda	2.88 (2.16, 3.85)	<0.001	2.66 (2.00, 3.55)	<0.001
Ghana	4.19 (3.08, 5.69)	<0.001	4.36 (3.18, 5.99)	<0.001
Nigeria	0.43 (0.27, 0.67)	<0.001	0.21 (0.12, 0.38)	<0.001
India	2.48 (1.84, 3.34)	<0.001	2.71 (2.02, 3.63)	<0.001
China	5.79 (3.96, 8.46)	<0.001	3.73 (2.51, 5.56)	<0.001

Abbreviations: RR, Relative risk. CI, Confidence intervals. RR was estimated from the log-linear regression models, i.e., the generalized linear regression models with the Poisson family, log-link function, and robust variance estimator. Estimates were obtained from the multivariable adjusted models, separately for anxiety and depressive symptoms, adjusted for all explanatory variables.

The country-specific analyses for each study site revealed some variations in the correlates. For instance, between countries, higher levels of anxiety symptoms among older adolescents (15–19 years) and those who visited a hospital in the past year were only observed in Burkina Faso and Uganda. Physical activity for five or more days a week was associated with lower levels of anxiety symptoms in Ghana, Nigeria, and Uganda, but higher levels in Burkina Faso. Furthermore, experiencing multiple injuries (≥2 times) increased the risk of anxiety symptoms among adolescents in Ghana, Tanzania, and China. A history of alcohol consumption had a strong positive association with anxiety symptoms among adolescents in Burkina Faso and Tanzania (**Table 4**).

**Table 4:** Correlates of anxiety symptoms among adolescents aged 10-19 years, separately for each country.

Characteristics	Burkina Faso (n = 1202)		Ethiopia (n = 1200)		Ghana (n = 1245)		Nigeria (n = 1264)	
	RR (95% CI)	p-value	RR (95% CI)	p-value	RR (95% CI)	p-value	RR (95% CI)	p-value
**Age** (15–19 years)	1.83 (1.19, 2.81)	0.01	9.74 (0.82, 115.19)	0.07	1.32 (0.96, 1.83)	0.09	0.89 (0.65, 1.23)	0.49
**Sex** (Female)	1.12 (0.76, 1.65)	0.57	1.63 (0.59, 4.48)	0.35	1.15 (0.89 1.49)	0.28	1.43 (1.04, 1.94)	0.03
**Education level** (Secondary or higher)	1.63 (1.06, 2.50)	0.03	0.97 (0.32, 2.93)	0.96	1.32 (0.99, 1.78)	0.06	1.39 (0.87, 2.23)	0.17
**Engaged in any work past year** (Yes)	0.55 (0.37, 0.84)	0.01	1.98 (0.91, 4.31)	0.09	1.14 (0.85, 1.52)	0.39	2.62 (1.96, 3.50)	<0.001
**MacArthur Scale of Subjective Social Status** (ref: High)								
Low	1.34 (0.56, 3.18)	0.51	0.23 (0.02, 3.59)	0.3	1.42 (0.93 2.17)	0.11	1.11 (0.52, 2.35)	0.79
Middle	0.74 (0.31, 1.77)	0.5	1.37 (0.22, 8.46)	0.74	1.24 (0.85, 1.80)	0.27	1.62 (0.85, 3.08)	0.15
**Visited primary care clinic past year** (Yes)	0.06 (0.01, 0.39)	0.004	1.79 (0.59, 5.43)	0.3	1.24(0.87, 1.77)	0.24	1.09 (0.59, 2.01)	0.79
**Visited a hospital past year** (Yes)	2.29 (1.54, 3.42)	<0.001	1.32 (0.65, 2.70)	0.44	1.08 (0.81, 1.42)	0.6	1.10 (0.80, 1.51)	0.54
**Visited a drug pharmacy or store past year** (Yes)	0.64 (0.28, 1.42)	0.27	–	–	0.89 (0.69, 1.13)	0.34	1.23 (0.92, 1.64)	0.16
**Have health insurance** (ref: No)								
Yes	1.40 (0.40, 4.94)	0.6	2.57 (1.28, 5.14)	0.01	1.00 (0.76, 1.32)	0.99	1.10 (0.67, 1.79)	0.71
Don’t know	1.34 (0.60, 3.00)	0.47	–	–	1.35 (0.43, 4.19)	0.61	0.19 (0.05, 0.68)	0.01
**Ever smoked cigarettes** (Yes)	1.79 (0.81, 3.96)	0.15	0.35 (0.05, 2.20)	0.26	0.79 (0.44, 1.40)	0.42	0.44 (0.07, 2.66)	0.37
**Ever drank alcohol** (Yes)	1.60 (1.11, 2.31)	0.01	0.49 (0.17, 1.39)	0.18	1.20 (0.92, 1.56)	0.18	1.25 (0.86, 1.81)	0.24
**Ever had sexual intercourse with a girl, woman, boy, or man** (Yes)	0.61 (0.36, 1.03)	0.06	2.21 (1.03, 4.73)	0.04	1.39 (1.03, 1.87)	0.03	0.79 (0.38, 1.64)	0.53
**Seriously injured multiple (≥** 2) **times past year** (Yes)	0.93 (0.31, 2.77)	0.9	6.58 (0.93, 46.5)	0.06	1.68 (1.24, 2.29)	0.001	1.11 (0.79, 1.56)	0.54
**Number of days physically active past week** (ref: None)								
1-4 days	3.16 (0.99, 10.02)	0.05	0.83 (0.18, 3.89)	0.82	0.58 (0.32, 1.04)	0.07	0.62 (0.40, 0.97)	0.03
5-7 days	4.17 (1.31, 13.25)	0.02	0.50 (0.11, 2.32)	0.38	0.38 (0.22, 0.64)	<0.001	0.35 (0.22, 0.56)	<0.001
**Body mass index** (ref: Normal)								
Underweight	1.11 (0.59, 2.11)	0.74	1.01 (0.34, 2.97)	0.98	1.36 (0.83, 2.23)	0.22	1.05 (0.65, 1.70)	0.85
Overweight/obese	1.33 (0.63, 2.83)	0.45	0.88 (0.31, 2.51)	0.82	1.46 (1.11, 1.93)	0.01	1.19 (0.71, 2.01)	0.5
**Characteristics**	**Tanzania (n = 1201)**		**Uganda (n = 1200)**		**China (n = 1291)**		**India (n = 1246)**	
	**RR (95% CI)**	**p-value**	**RR (95% CI)**	**p-value**	**RR (95% CI)**	**p-value**	**RR (95% CI)**	**p-value**
**Age** (15–19 years)	1.28 (0.64, 2.52)	0.48	1.50 (1.07, 2.11)	0.02	1.01 (0.93, 1.09)	0.82	12.06 (1.45, 100.14)	0.02
**Sex** (Female)	2.26 (1.25, 4.10)	0.01	1.11 (0.80, 1.52)	0.54	1.11 (0.92, 1.35)	0.26	0.59 (0.26, 1.33)	0.2
**Education level** (Secondary or higher)	1.23 (0.68, 2.25)	0.5	1.18 (0.77, 1.80)	0.46	–		0.14 (0.02, 1.15)	0.07
**Engaged in any work past year** (Yes)	1.41 (0.79, 2.52)	0.25	1.03 (0.68, 1.57)	0.88	–		0.61 (0.22, 1.69)	0.34
**MacArthur Scale of Subjective Social Status** (ref: High)								
Low	–		1.72 (0.42, 6.98)	0.45	1.09 (0.74, 1.63)	0.64	8.74 (1.53, 50.04)	0.02
Middle	–		0.85 (0.21, 3.47)	0.82	0.90 (0.63, 1.29)	0.57	2.43 (0.56. 10.61)	0.24
**Visited primary care clinic past year** (Yes)	0.55 (0.17, 1.73)	0.3	1.48 (1.07, 2.06)	0.02	0.96 (0.77, 1.19)	0.69	0.93 (0.29, 2.97)	0.9
**Visited a hospital past year** (Yes)	1.29 (0.74, 2.25)	0.37	1.38 (0.97, 1.96)	0.08	1.11 (0.91, 1.35)	0.32	1.61 (0.68, 3.81)	0.28
**Visited a drug pharmacy or store past year** (Yes)	0.66 (0.24, 1.82)	0.42	2.04 (1.52, 2.76)	<0.001	1.12 (0.93, 1.36)	0.22	1.30 (0.41, 4.11)	0.65
**Have health insurance** (ref: No)								
Yes	0.41 (0.15, 1.15)	0.09	–		0.82 (0.62, 1.09)	0.18	1.08 (0.46, 2.54)	0.86
Don’t know	–		–		0.94 (0.74, 1.21)	0.64	–	
**Ever smoked cigarettes** (Yes)	1.66 (0.68, 4.02)	0.26	2.53 (0.38, 17.01)	0.34	1.22 (0.81, 1.84)	0.35	–	
**Ever drank alcohol** (Yes)	2.22 (1.10, 4.47)	0.03	0.47 (0.15, 1.46)	0.19	1.07 (0.85, 1.36)	0.56	–	
**Ever had sexual intercourse with a girl, woman, boy, or man** (Yes)	1.83 (0.91, 3.67)	0.09	0.89 (0.55, 1.45)	0.66	1.64 (1.05, 2.57)	0.03	28.67 (4.25, 193.37)	0.001
**Seriously injured multiple (≥** 2) **times past year** (Yes)	7.99 (3.36, 19.04)	<0.001	1.36 (0.77, 2.39)	0.3	–		7.59 (1.86, 31.07)	0.01
**Number of days physically active past week** (ref: None)								
1-4 days	0.91 (0.50, 1.63)	0.75	0.74 (0.51, 1.08)	0.12	0.92 (0.65, 1.30)	0.63	0.79 (0.29, 2.10)	0.63
5-7 days	0.66 (0.33, 1.32)	0.24	0.19 (0.11, 0.35)	<0.001	0.83 (0.58, 1.19)	0.31	0.99 (0.37, 2.68)	0.99
**Body mass index** (ref: Normal)								
Underweight	0.60 (0.19, 1.91)	0.39	1.59 (1.03, 2.47)	0.04	1.27 (0.19, 8.48)	0.81	1.21 (0.48, 3.03)	0.69
Overweight/obese	0.50 (0.23, 1.09)	0.08	0.64 (0.33, 1.24)	0.19	0.93 (0.72, 1.21)	0.59	1.80 (0.65, 4.99)	0.26

Abbreviations: RR, Relative risk. CI, Confidence intervals. RR was estimated from the log-linear regression models, i.e., the generalized linear regression models with the Poisson family, log-link function, and robust variance estimation. Estimates were obtained from the multivariable adjusted models, separately for anxiety and depressive symptoms, adjusted for all explanatory variables, across each study country. Some estimates are very small, therefore presented by a dash (-). Wide CIs for some variables are because of small number of cases.

### Correlates of depressive symptoms

Older adolescence, being female, and engaging in any work in the past year were linked to an increased risk of experiencing depressive symptoms. Adolescents who reported low perceived social status compared to high, and visited a primary care clinic, hospital, and drug pharmacy/ store in the past year, compared to those who did not, also had a significantly higher risk of experiencing depressive symptoms. Likewise, adolescents who reported ever drinking alcohol and who had been seriously injured twice or more in the past year were at a significantly increased risk of experiencing depressive symptoms than those who had not. Furthermore, regarding depressive symptoms, a similar pattern to anxiety symptoms was observed for the association with the study country. Adolescents from Burkina Faso, Uganda, Ghana, Nigeria, and China showed strong positive associations, while Ethiopia and India exhibited inverse protective associations (**Table 3**).

The country-specific analyses for each study site also revealed some variations in the correlates of depressive symptoms. Regarding correlates of depressive symptoms, between countries, strong positive associations were also observed in Burkina Faso, Ethiopia, and Ghana among older adolescents, those who visited a hospital in the past year preceding the surveys, and those who had health insurance. Female adolescents in Nigeria had a significantly higher likelihood of depressive symptoms than males, while in other countries there were no significant sex differences. Substance use, particularly a history of alcohol consumption, was positively associated with depressive symptoms, only among adolescents in Tanzania and China (**Table 5**).

**Table 5:** Correlates of depressive symptoms among adolescents aged 10-19 years, separately for each country.

Characteristics	Burkina Faso (n = 1202)		Ethiopia (n = 1200)		Ghana (n = 1245)		Nigeria (n = 1264)	
	RR (95% CI)	p-value	RR (95% CI)	p-value	RR (95% CI)	p-value	RR (95% CI)	p-value
**Age** (15–19 years)	3.15 (1.94, 5.11)	<0.001	2.29 (0.28, 18.75)	0.44	1.47 (1.08, 2.00)	0.02	0.83 (0.61, 1.13)	0.23
**Sex** (Female)	0.84 (0.54, 1.30)	0.44	2.19 (0.64, 7.48)	0.21	1.11 (0.88, 1.40)	0.39	1.50 (1.10, 2.04)	0.01
**Education level** (Secondary or higher)	0.80 (0.49, 1.30)	0.37	1.42 (0.28, 7.15)	0.67	1.12 (0.85, 1.48)	0.41	1.63 (1.00, 2.65)	0.05
**Engaged in any work past year** (Yes)	0.63 (0.41, 0.96)	0.03	1.77 (0.63, 4.98)	0.28	1.18 (0.91, 1.53)	0.21	2.17 (1.61, 2.92)	<0.001
**MacArthur Scale of Subjective Social Status** (ref: High)								
Low	6.07 (0.97, 38.15)	0.05	–		1.20 (0.85, 1.69)	0.3	1.09 (0.55, 2.17)	0.81
Middle	3.30 (0.53, 20.58)	0.2	0.65 (0.12, 3.48)	0.61	0.84 (0.61, 1.15)	0.28	1.44 (0.80, 2.58)	0.22
**Visited primary care clinic past year** (Yes)	0.41 (0.17, 1.01)	0.05	1.08 (0.14, 8.58)	0.94	0.94 (0.65, 1.36)	0.74	1.53 (0.90, 2.59)	0.12
**Visited a hospital past year** (Yes)	2.93 (1.78, 4.83)	<0.001	3.56 (1.43, 8.84)	0.01	0.92 (0.70, 1.22)	0.58	1.20 (0.88, 1.64)	0.25
**Visited a drug pharmacy or store past year** (Yes)	1.53 (0.78, 2.99)	0.21	1.84 (0.29, 11.55)	0.51	1.09 (0.88, 1.37)	0.43	1.37 (1.04, 1.82)	0.03
**Have health insurance** (ref: No)								
Yes	4.17 (2.12, 8.22)	<0.001	2.91 (1.22, 6.93)	0.02	0.83 (0.65, 1.06)	0.14	0.99 (0.59, 1.70)	0.99
Don’t know	1.23 (0.42, 3.65)	0.71	0.58 (0.06, 5.46)	0.63	0.65 (0.11, 3.76)	0.63	0.50 (0.23, 1.12)	0.09
**Ever smoked cigarettes** (Yes)	1.13 (0.42, 3.07)	0.81	0.60 (0.05, 6.95)	0.69	1.55 (1.02, 2.36)	0.04	0.35 (0.06, 2.25)	0.27
**Ever drank alcohol** (Yes)	0.91 (0.56, 1.50)	0.72	1.12 (0.34, 3.67)	0.85	1.27 (0.99, 1.64)	0.06	1.09 (0.73, 1.63)	0.68
**Ever had sexual intercourse with a girl, woman, boy, or man** (Yes)	0.78 (0.46, 1.31)	0.34	1.09 (0.38, 3.17)	0.87	1.01 (0.74, 1.36)	0.97	1.52 (0.91, 2.54)	0.11
**Seriously injured multiple (≥** 2**) times past year** (Yes)	1.91 (0.84, 4.31)	0.12	–		1.52 (1.14, 2.04)	0.01	1.03 (0.73, 1.46)	0.87
**Number of days physically active past week** (ref: None)								
1-4 days	1.64 (0.61, 4.36)	0.33	0.43 (0.09, 1.95)	0.28	0.68 (0.36, 1.31)	0.25	0.61 (0.38, 0.96)	0.03
5-7 days	2.38 (0.91, 6.30)	0.08	0.39 (0.09, 1.63)	0.2	0.46 (0.26, 0.83)	0.01	0.48 (0.31, 0.75)	0.001
**Body mass index** (ref: Normal)								
Underweight	0.38 (0.12, 1.24)	0.11	0.71 (0.15, 3.35)	0.67	1.07 (0.65, 1.76)	0.79	1.40 (0.81, 2,13)	0.12
Overweight/obese	1.19 (0.50, 2.83)	0.7	0.84 (0.25, 2.86)	0.79	1.08 (0.82, 1.43)	0.58	1.22 (0.74, 1.99)	0.44
**Characteristics**	**Tanzania (n = 1201)**		**Uganda (n = 1200)**		**China (n = 1291)**		**India (n = 1246)**	
	**RR (95% CI)**	**p-value**	**RR (95% CI)**	**p-value**	**RR (95% CI)**	**p-value**	**RR (95% CI)**	**p-value**
**Age** (15–19 years)	1.04 (0.55, 1.99)	0.9	1.54 (1.10, 2.14)	0.01	1.08 (0.96, 1.19)	0.2	0.50 (0.16, 1.55)	0.23
**Sex** (Female)	1.02 (0.58, 1.78)	0.95	0.93 (0.69, 1.25)	0.64	1.08 (0.84, 1.39)	0.54	0.99 (0.35, 2.79)	0.98
**Education level** (Secondary or higher)								
**Engaged in any work past year** (Yes)	0.96 (0.55, 1.67)	0.88	0.99 (0.63, 1.56)	0.98	–		4.55 (1.14, 18.17)	0.03
**MacArthur Scale of Subjective Social Status** (ref: High)	1.71 (0.97, 3.04)	0.07	0.83 (0.53, 1.29)	0.41	–		0.13 (0.01, 7.61)	0.325
Low								
Middle	–		–		1.67 (0.93, 2.99)	0.08	–	
**Visited primary care clinic past year** (Yes)	–		–		1.08 (0.62, 1.88)	0.78	0.49 (0.14, 1.75)	0.27
**Visited a hospital past year** (Yes)	0.71 (0.29, 1.77)	0.47	1.71 (1.25, 2.33)	0.001	1.25 (0.97, 1.62)	0.09	0.71 (0.10, 4.99)	0.73
**Visited a drug pharmacy or store past year** (Yes)	0.95 (0.55, 1.64)	0.86	1.33 (0.94, 1.87)	0.1	1.39 (1.08, 1.79)	0.01	3.32 (1.04, 10.59)	0.04
**Have health insurance** (ref: No)	0.41 (0.13, 1.28)	0.13	1.97 (1.48, 2.61)	<0.001	1.13 (0.88, 1.45)	0.33	0.71 (0.09, 5.70)	0.75
Yes								
Don’t know	0.97 (0.45, 2.09)	0.93	–		0.83 (0.59. 1.16)	0.28	0.94 (0.21, 4.16)	0.94
**Ever smoked cigarettes** (Yes)	–		–		0.71 (0.52, 0.96)	0.03	–	–
**Ever drank alcohol** (Yes)	1.05 (0.44, 2.53)	0.91	–		1.13 (0.67, 1.91)	0.65		
**Ever had sexual intercourse with a girl, woman, boy, or man** (Yes)	3.68 (1.71, 7.91)	0.001	0.93 (0.32, 2.73)	0.9	1.47 (1.12, 1.93)	0.01		
**Seriously injured multiple (≥** 2**) times past year** (Yes)	1.69 (0.83, 3.41)	0.15	0.72 (0.42, 1.23)	0.24	1.30 (0.64, 2.64)	0.46	166.91 (5.45, 5113.6)	0.003
**Number of days physically active past week** (ref: None)	1.39 (0.39, 4.85)	0.61	1.46 (0.85, 2.50)	0.17	–		–	
1-4 days								
5-7 days	0.90 (0.49, 1.67)	0.75	0.58 (0.41, 0.81)	0.002	1.27 (0.75, 2.18)	0.38	0.09 (0.03, 0.28)	<0.001
**Body mass index** (ref: Normal)	0.81 (0.41, 1.61)	0.56	0.09 (0.04, 0.17)	<0.001	1.13 (0.66, 1.93)	0.67	0.94 (0.28, 3.13)	0.91
Underweight								
Overweight/obese	0.52 (0.16, 1.72)	0.28	1.52 (0.95, 2.43)	0.08	4.32 (1.70, 11.01)	0.002	1.35 (0.37, 4.91)	0.65

Abbreviations: RR, Relative risk. CI, Confidence intervals. RR was estimated from the log-linear regression models, i.e., the generalized linear regression models with the Poisson family, log-link function, and robust variance estimation. Estimates were obtained from the multivariable adjusted models, separately for anxiety and depressive symptoms, adjusted for all explanatory variables, across each study country. Some estimates are very small, therefore presented by a dash (-). Wide CIs for some variables are because of a small number of cases.

## Discussion

In this multi-country cross-sectional study, we determined the prevalence and correlates of anxiety and depressive symptoms among adolescents in six SSA countries, China, and India. Overall, the prevalence of anxiety and depressive symptoms was 11.3% and 9.9%, respectively, and was common in China, Ghana, and Nigeria. The lowest prevalence rates were found in India and Ethiopia. The risk of these mental health outcomes was higher for older adolescents (15–19 years), females, those who worked in the past year, with perceived low social status, and with a history of alcohol consumption. Significant associations of these correlates varied across SSA and Asia, for both anxiety and depressive symptoms.

The overall prevalence of anxiety and depressive symptoms in this study varied from a systematic review of observational epidemiological studies of mental health problems among adolescents in 16 SSA countries [2], which reported higher prevalence of anxiety (29.8%) and depressive (26.9%) symptoms. In contrast, our findings are slightly higher than the estimates (5.5% for anxiety and 3.1% for depression) from 26 LMICs (including SSA, northern African, and Asian countries) [3]. These differences could be explained by variations in data collection tools, methods, and study populations. While our study used the PHQ-2 and GAD-2, the study conducted across 26 LMICs used data collected from household surveys using the Child Functioning Module, a tool that specifically captures functional difficulties, including depression and anxiety among adolescents [3].

In our study, the highest prevalence of anxiety and depressive symptoms was found among young, in-school adolescents from China. These findings are similar to a cross-sectional study in China conducted during the post-coronavirus disease (COVID-19) pandemic era [5], reporting higher prevalence of anxiety (26.9%) and depression symptoms (25.6%) among adolescents. Multiple reasons have been cited for the increased mental health problems post-COVID-19, including fewer social interactions due to school closures, less physical activity, irregular sleep patterns, and fear of the pandemic [16]. The current study was also conducted during the COVID-19 pandemic, with countries having different control measures, such as with or without lockdowns, which may explain the observed variations.

The prevalence of anxiety and depressive symptoms was the lowest among adolescents from India. Although the National Mental Health survey (NMHS) in India, conducted in 2016, found a prevalence of 2.6% for depressive disorders among youth, other independent studies have found higher prevalence of depression, ranging from 6.6% to 54% [17,18]. Our study results differ from those of prior research, presumably due to variations in assessment tools, study settings, cultural factors, sampled populations, or healthcare system factors [19]. While our study assessed anxiety and depressive symptoms using the brief versions of the PHQ and GAD tools, other studies have used complete versions of the same tools, that is, the PHQ-9 and GAD-7. In India, population diversity across states has also been reported as a factor for data variations [19].

Within SSA countries, the highest prevalence was among adolescents from Ghana, Nigeria, and Uganda. Differences in adolescent characteristics or socio-cultural practices across these countries could explain the high prevalence, since correlates such as older age among adolescents, female gender, lower socio-economic status, and risk behaviors have commonly been associated with higher prevalence of anxiety and depressive symptoms [20]. Risk behaviors such as a history of tobacco smoking, alcohol consumption, and being seriously injured twice or more times in the past year were also common among adolescents in Ghana and Nigeria, compared to other countries. A recent comparable cross-sectional study from primary care clinics in Ghana and Nigeria reported a 16% burden of mental health disorders among adolescents, with the most common disorder being depression [21]. Compared to all other countries, the highest proportion of adolescents from Uganda had a perceived low social status, where high poverty has been reported and associated with mental health outcomes, especially in the rural areas [22]. Similarly, female gender, unemployment, and co-morbid psychiatric disorders have been identified as significant correlates of anxiety symptoms among adolescents in Uganda [22]. Ethiopia had the lowest prevalence of anxiety and depressive symptoms across SSA countries, which contrasts with national estimates of 12–25% prevalence of adolescent mental illnesses, with around 14% of the youth having depression and 10% having anxiety in 2022 [23]. In Tanzania, the rates of anxiety (5%) and depressive symptoms (3.1%) in this study were consistent with a school-based survey that reported 7% of students feeling sad and lonely/depressed daily [24]. Yet, evidence on the anxiety and depression burden among adolescents in Tanzania remains scant.

Previous studies have reported a greater risk of anxiety and depression symptoms among females, older adolescents, those with higher levels of school grades, engaged in irregular physical activity, urban residents, consumed alcohol, with a history of tobacco smoking, and experiencing bullying, violence, and food insecurity [6,20,25]. Country-specific analyses showed some variations in these correlates across countries in SSA and Asia. Higher prevalence among older adolescents could be attributed to their greater social needs for peer relationships, social acceptance, and physical changes [6,25]. Older adolescents in Burkina Faso and Uganda, but not in other countries, showed a higher likelihood of experiencing anxiety symptoms, suggesting country-specific factors such as political instability, insecurity, high levels of violence or conflict [22,26] Female adolescents in this study had a higher risk of experiencing anxiety and depressive symptoms than males. This finding has been previously reported [27] in studies that found female adolescents being vulnerable to stress, expressed feelings of guilt and dissatisfaction, sadness, self-blame, and depressed mood, more than their male counterparts [27], while males are socialized to hide their feelings, as per gender or societal norms [28]. Gender differences in depressive symptoms have also been linked to higher rumination in females than males [28].

Adolescents with perceived low social status were also at a higher risk of anxiety and depressive symptoms. A study from Uganda reported that a low perceived social status was associated with depression [29]. Poverty has also been identified as a psychosocial stressor previously [3]. The high risk of mental disorders could be associated with other poverty-related factors, such as food insecurity and a lack of resources to access services [9]. This finding might explain the observed positive association between engaging in any work in the past year with the increased risk of anxiety symptoms among adolescents from Nigeria in this study.

In our study, adolescents who reported being engaged in risky behaviors, such as alcohol consumption, ever having sexual intercourse, and being seriously injured twice or more times in the past year, had a higher risk of experiencing anxiety and depressive symptoms. Alcohol consumption and smoking cigarettes have been commonly reported risk factors for mental health conditions among adolescents [6,25]. In our study, only a small proportion of adolescents had a history of tobacco smoking (3.1%), which could explain why smoking was not significantly associated with anxiety or depressive symptoms in the overall all adolescent population. Nevertheless, smoking was associated with a higher likelihood of depressive symptoms among adolescents in Ghana and a lower likelihood among those in China.A cross-sectional study in LMICs [30] suggests that multiple serious physical injuries could lead to disability and damage the nervous system, which in turn increases depressive symptoms. In this study, adolescents with multiple injuries from Ghana, India, and Tanzania revealed a higher likelihood of anxiety symptoms. The association between a history of sexual intercourse and poor mental health has been studied previously [31]; it has been attributed to individual and contextual factors such as trying to fit in, experimentation, affection seeking, or anger among adolescents. On the other hand, higher prevalence of anxiety and depression has also been reported among those with multiple sexual partners and experiences of sexual violence [31].

In this study, being physically active for 5–7 days in the past week significantly decreased the risk of anxiety and depressive symptoms. These findings concur with those of previous studies, which link physical activity to increased social and personal skills and improved self-esteem, all of which can positively impact mental health outcomes [32]. In contrast, adolescents from Burkina Faso who reported to be physically active for 5–7 days in the past week had a higher risk of experiencing anxiety symptoms compared to other countries. This may suggest that the most vulnerable adolescents were physically active in the past week, rather than indicating a direct correlation between physical activity and anxiety symptoms. Adolescents with overweight/obesity are also vulnerable to negative self-perceptions and anxiety or depressive symptoms. A recent cohort study from three LMICs described the prevalence of weight-related stigma among adolescents [33]; negative self-perceptions, dissatisfaction, anxiety, and depressive symptoms were reported, especially by females. This finding highlights the need for promoting a healthy lifestyle among adolescents, such as programs to increase physical activities, access to healthy food options, and mental health care.

### Strengths and limitations

Our results provide needed knowledge on the prevalence and factors associated with anxiety and depressive symptoms among adolescents in LMICs using data from multiple sites, collected using a standardized questionnaire across countries. The findings may be generalizable to adolescent populations with similar demographic profiles and geographical settings due to the large sample size of our study. Except in Nigeria and China, data collection was community-based. Findings from this study address the knowledge gaps on anxiety and depression among adolescents in SSA countries, India, and China. This study also had several limitations; the prevalence of anxiety and depressive symptoms could be under-estimated due to under-reporting related to stigma among adolescents. The use of a brief questionnaire, PHQ-4, could also limit the capture of clinically, culturally, and contextually appropriate symptoms. There could also be recall bias in reporting visits to clinics or hospitals, and the number of days of physical activity, which may have affected their association with anxiety and depressive symptoms.

## Conclusion and implications

Across six SSA and two Asian countries, anxiety and depressive symptoms are common among adolescents from China, Ghana, and Nigeria and relatively rare in adolescents from India and Ethiopia. These symptoms of mental health issues are associated with socio-demographic characteristics and behaviors. School- and community-based interventions addressing these factors could reduce the burden of mental health problems in adolescence. Future mixed-methods studies are crucial to complement the quantitative findings with an in-depth understanding of socioeconomic characteristics, behaviors, and contextual issues affecting young people’s mental health. Such studies could provide further insights into individuals’ views on mental health as well as the societal and cultural norms shaping their perceptions, which may inform context-specific interventions. We also recommend longitudinal studies to monitor trends, establish the dose-effect relationships for the observed associations, and generate evidence to inform policy decisions and interventions to improve adolescent health and well-being.

## Supporting information

S1 Checklist Inclusivity in Global Health Research questionnaire. (PDF)
